# LDL Receptor Pathway Regulation by miR-224 and miR-520d

**DOI:** 10.3389/fcvm.2020.00081

**Published:** 2020-05-22

**Authors:** Alessandro G. Salerno, Coen van Solingen, Elena Scotti, Amarylis C. B. A. Wanschel, Milessa S. Afonso, Scott R. Oldebeken, Westley Spiro, Peter Tontonoz, Katey J. Rayner, Kathryn J. Moore

**Affiliations:** ^1^Leon H. Charney Division of Cardiology, NYU Cardiovascular Research Center, Department of Medicine, New York University School of Medicine, New York, NY, United States; ^2^Howard Hughes Medical Institute and Department of Pathology and Laboratory Medicine, University of California, Los Angeles, Los Angeles, CA, United States; ^3^Department of Biochemistry, Microbiology and Immunology, University of Ottawa Heart Institute, Ottawa, ON, Canada; ^4^Department of Cell Biology, New York University School of Medicine, New York, NY, United States

**Keywords:** noncoding RNA, low-density lipoprotein, cholesterol, PCSK9, IDOL, HMGCR

## Abstract

MicroRNAs (miRNA) have emerged as important post-transcriptional regulators of metabolic pathways that contribute to cellular and systemic lipoprotein homeostasis. Here, we identify two conserved miRNAs, miR-224, and miR-520d, which target gene networks regulating hepatic expression of the low-density lipoprotein (LDL) receptor (LDLR) and LDL clearance. *In silico* prediction of miR-224 and miR-520d target gene networks showed that they each repress multiple genes impacting the expression of the LDLR, including the chaperone molecules PCSK9 and IDOL that limit LDLR expression at the cell surface and the rate-limiting enzyme for cholesterol synthesis HMGCR, which is the target of LDL-lowering statin drugs. Using gain- and loss-of-function studies, we tested the role of miR-224 and miR-520d in the regulation of those predicted targets and their impact on LDLR expression. We show that overexpression of miR-224 or miR-520d dose-dependently reduced the activity of *PCSK9, IDOL*, and *HMGCR* 3′-untranslated region (3′-UTR)-luciferase reporter constructs and that this repression was abrogated by mutation of the putative miR-224 or miR-520d response elements in the *PCSK9, IDOL*, and *HMGCR* 3′-UTRs. Compared to a control miRNA, overexpression of miR-224 or miR-520d in hepatocytes inhibited *PCSK9, IDOL*, and *HMGCR* mRNA and protein levels and decreased PCSK9 secretion. Furthermore, miR-224 and miR-520d repression of *PCSK9, IDOL*, and *HMGCR* was associated with an increase in LDLR protein levels and cell surface expression, as well as enhanced LDL binding. Notably, the effects of miR-224 and miR-520d were additive to the effects of statins in upregulating LDLR expression. Finally, we show that overexpression of miR-224 in the livers of *Ldlr*^+/−^ mice using lipid nanoparticle-mediated delivery resulted in a 15% decrease in plasma levels of LDL cholesterol, compared to a control miRNA. Together, these findings identify roles for miR-224 and miR-520d in the posttranscriptional control of LDLR expression and function.

## Introduction

Elevated levels of apoB-containing lipoproteins, particularly low-density lipoprotein (LDL), are a major risk factor for atherosclerotic cardiovascular diseases (CVDs). Plasma levels of LDL cholesterol (LDL-C) are largely controlled by the balance of hepatic lipoprotein synthesis, dietary cholesterol intake, and LDL particle clearance by the liver. The LDL receptor (LDLR) is highly expressed on the plasma membrane of hepatocytes and binds LDL particles to clear them from the circulation (1, 2). Thus, factors that affect LDLR expression and/or activity influence circulating LDL-C levels and the resultant risk of atherosclerosis. The pharmacologic reduction of LDL-C using statins is currently the most widespread clinical intervention in the management of atherosclerosis. This class of small molecules interferes with cholesterol synthesis by inhibiting HMG-CoA reductase (HMGCR), a rate-limiting enzyme in the cholesterol biosynthetic pathway, leading to a reduction in intracellular cholesterol levels that triggers translocation of the SREBP2 transcription factor to the nucleus and transcriptional activation of the *LDLR* gene. The resultant upregulation of LDLR expression by the liver increases clearance of LDL particles and decreases plasma levels of LDL-C, with 1 mmol/L (~40 mg/dL) of LDL-C being associated with 22% reduction in major vascular events and a 10% reduction in mortality. Despite the efficacy of LDL-C lowering by statins, optimal therapy in clinical trials achieved only a 45% reduction in risk of major coronary events, leading to the search for other LDL-lowering interventions. A better understanding of the posttranscriptional mechanisms regulating LDLR expression and LDL-C clearance may enable the discovery of novel cholesterol-lowering drugs.

Proprotein convertase subtilisin/kexin type 9 (PCSK9) is a secreted protein that interrupts the recycling of the LDLR by diverting it for lysosomal degradation after receptor-mediated endocytosis of LDL particles (3). Gain-of-function mutations in PCSK9 cause autosomal dominant hypercholesterolemia, with elevated LDL-C and associated premature coronary artery disease (CAD) (3, 4). Conversely, individuals with either complete loss-of-function alleles (no detectable PCSK9 levels) or partial loss-of-function alleles (truncation or missense mutations) typically have a lifelong decrease in LDL-C (28–40%) and lower risk of CAD (~40–90%) (5–7). PCSK9 expression is induced by SREBP2 and is therefore increased upon inhibition of HMGCR by statins, resulting in an attenuation of LDLR expression induced by statin therapy, thereby blunting the overall effectiveness of statins on LDL lowering. Monoclonal antibodies against PCSK9 are effective in lowering LDL-C and are currently used for the treatment of individuals with familial hypercholesterolemia, who are refractory to statins due to mutations in the LDLR (8).

A second chaperone protein that regulates LDLR cell surface expression is the inducible degrader of the LDLR (IDOL), an E3-ubiquitin ligase that promotes ubiquitination of the LDLR, thereby marking it for lysosomal degradation (9). In contrast to PCSK9, whose levels are stimulated by low sterol levels, IDOL is induced when sterol levels are high to prevent the uptake of further cholesterol from the periphery. IDOL is transcriptionally regulated by the liver X receptors (LXRs) which coordinate the cellular response to cholesterol excess by activating the transcription of genes such as *ABCA1, ABCG1*, and *APOE*. PCSK9 and IDOL have been shown to function in non-redundant complementary pathways and could thus be targeted in concert to prevent LDLR degradation (10). Together, these discoveries highlight how expanding our understanding of how the LDLR is regulated at the posttranscriptional level can enable the further discovery of novel classes of cholesterol-lowering agents that go beyond transcriptional activation of LDLR.

MicroRNAs (miRNAs) are posttranscriptional regulators of gene expression that have emerged as important fine tuners of lipid metabolism pathways. miRNAs are endogenous ~22-nucleotide noncoding RNAs that regulate target gene expression by imperfect base pairing to the 3′-untranslated regions (3′-UTRs) of mRNAs, resulting in the inhibition of translation and/or degradation of the transcript (11–13). A single miRNA can regulate the expression of multiple target genes in concert, thereby providing a mechanism for synchronized inhibition of functional gene networks (14). Thus, although the effects of a miRNA on any single gene can be modest (often in the order of 10–20%), its cumulative effects can be profound. This is exemplified by the discovery of miR-33 as a central regulator of cellular lipid metabolism pathways. miR-33a and miR-33b are positioned within intronic sequences of the *SREBF2* and *SREBF1* genes, respectively, and are co-transcribed with their host genes. miR-33a/miR-33b boosts cellular lipid levels by repressing genes that promote cholesterol efflux (*ABCA1, ABCG1, NPC1, OSBPL6, ATG5*, and *LAMP1*), reverse cholesterol transport (*ABCA1, ATP8B1*, and *ABCG11*), and fatty acid oxidation (*CROT, CPT1A, HADHB*, and *PRKAA1*) (15–19). Delivery of miR-33 inhibitors in mice and nonhuman primates increases plasma levels of high-density lipoprotein (HDL) cholesterol by 40–50% and promotes reverse cholesterol transport. In addition to miR-33, several other miRNAs have been reported to modulate HDL cholesterol transport through regulation of ABCA1-dependent cholesterol efflux [e.g., miR-27, miR-144, miR-145, miR-223, and miR-758 (20–24)] or hepatic HDL-C uptake via SR-B1 [e.g., miR-96, miR-185, and miR-223 (25, 26)].

miRNAs that regulate plasma levels of LDL-C by direct targeting of LDLR mRNA have also recently been reported, including miR-27a/miR-27b, miR-128, miR-130b, miR-184a, miR-185, and miR-301b (27–30). However, the putative role of miRNAs in controlling the expression of proteins that indirectly regulate LDLR cell surface expression, such as PCSK9, IDOL, and HMGCR, has been less well-studied. Here, we investigate the function of two conserved miRNAs, miR-224 and miR-520d, predicted to target overlapping gene networks regulating LDL homeostasis. We demonstrate that miR-224 and miR-520d target both PCSK9 and IDOL, chaperone proteins that promote the degradation of LDLR, as well as targeting HMGCR, an enzyme whose inhibition increases *LDLR* expression. Functional studies in hepatocytes revealed that miR-224 and miR-520d reduce mRNA and proteins of PCSK9, IDOL, and HMGCR, resulting in increased LDLR cell surface expression and binding of LDL. These data demonstrate roles for miR-224 and miR-520d in controlling LDLR cell surface expression and LDL homeostasis.

## Experimental Procedures

### Cell Culture

HepG2 and HEK-293T cells were obtained from the American Type Tissue Collection, authenticated with standard American Type Tissue Collection methods (morphology check under a microscope and growth curve analysis) and regularly tested for mycoplasma contamination. Cells were maintained in Dulbecco's modified Eagle medium (DMEM, Corning) containing 10% fetal bovine serum (FBS, Life Technologies) and 1% penicillin–streptomycin (pen-strep, Life Technologies). HepG2-LDLR-GFP cells were grown as previously described (9). All cells were cultured in a humidified incubator at 37°C and 5% CO_2_.

### Luciferase Assay

HEK-293T cells, at a density of 10^5^ cells per well, were co-transfected with 3′-UTR luciferase reporter plasmids (SwitchGear Genomics) using Lipofectamine 2000 transfection reagent (Invitrogen) and miRNA mimics (40 nM final concentration, Dharmacon) in 24-well plates. Luciferase activity was measured after 48 h with the Dual Luciferase Assay System (Promega). *Firefly luciferase* activity was normalized to the corresponding *Renilla luciferase* activity and plotted as a percentage of the control cells co-transfected with the corresponding concentration of control mimic.

### Mutagenesis Assembly

Mutagenesis of the miRNA-224 and miRNA-520d response elements (MREs) in 3′-UTR luciferase reporter plasmids for *PCSK9, IDOL*, and *HMGCR* was carried out according to the instructions supplied with the QuikChange XL II site-directed mutagenesis kit (Stratagene) using the primers listed in Supplementary Table 1. The sequences of the mutated fragments were confirmed by Sanger sequencing.

### RNA Isolation and Quantitative RT-PCR

Total RNA was isolated with TRIzol Reagent (Invitrogen) and Direct-zol RNA MiniPrep Columns (Zymo Research). RNA was reverse transcribed using an iScript™ cDNA Synthesis Kit (Bio-Rad), according to the manufacturer's protocol. qRT-PCR analysis was conducted using iQ SYBR Green Supermix (Bio-Rad) in a Mastercycler PCR machine (Eppendorf). miRNA quantification was performed in with the miScript SYBR Green PCR Kit (Qiagen) using the Qiagen miScript Primers in a Mastercycler PCR machine. qRT-PCR and miScript primers are listed in Supplementary Table 2. Fold change in mRNA and miRNA expression was calculated with the comparative cycle method (2^−ΔΔCt^) and normalized to the housekeeping genes [*GAPDH* for mRNA; RNU-6 (*U6*) for miRNA].

### Transfection of Hepatic Cells With siRNA, miRNA Mimics, and Inhibitors

Human hepatic cells (HepG2) were plated at a density of 10^6^ cells per well on a six-well cell culture plates; were transfected with control, miRNA mimics or inhibitors (80 nM, Dharmacon), or siRNA (40 nM, Dharmacon) using Lipofectamine® RNAi MAX (Invitrogen); and subsequently stimulated with GW3965 (1 μM, Sigma-Aldrich) or simvastatin (5 μM, Sigma-Aldrich) for an additional 24 h. For cells treated with simvastatin, the medium was replaced with a cholesterol-depleted medium [DMEM containing 5% bovine lipoprotein-deficient serum (LPDS)], 24 h before simvastatin treatment. After treatment, cells were harvested and lysed with either TRIzol Reagent for RNA expression analysis or radioimmunoprecipitation assay buffer (RIPA, Abcam) for protein expression analysis.

### Western Blot Analysis and ELISA

PCSK9 (CY-P1037, MBL International), IDOL [as described in (9)], HMGCR (ab174830, Abcam), LDLR (1007665, Cayman Chemical), tubulin (T6074, Sigma-Aldrich), SREBP2 (Abcam, ab30682), and β-actin (sc-47778, Santa Cruz Biotechnology) antibodies were used for western blotting. To detect IDOL protein levels, cells were pretreated with MG-132 (10 μM, Sigma-Aldrich). Protein bands were visualized using the Odyssey Infrared Imaging System (LI-COR Biotechnology). Densitometry analysis of the gels was carried out using ImageJ software from the NIH (http://rsbweb.nih.gov/ij/). To measure secreted PCSK9 levels, the supernatants were used in a PCSK9 enzyme-linked immunosorbent assay (ELISA, R&D Systems).

### Immunofluorescence Microscopy

HepG2 or HepG2-LDLR-GFP cells resuspended in 10% LPDS were plated in chamber slides (Lab-Tek II, Thermo Scientific) and transfected with miR-224 or miR-520d mimics (80 nM) and stimulated with GW3965 (1 μM) or simvastatin (5 μM) for an additional 24 h. For LDLR internalization imaging in HepG2, the cells were incubated with diI-LDL (2.5 μg/mL, Biomedical Technologies) for 30 min and washed with cold PBS. The cells were fixed with 4% PFA in PBS. Cells treated with diI-LDL were incubated with DAPI (D-9542, Sigma Aldrich) for 10 min. After washing, cells were mounted with a mounting medium for fluorescence (H-1000, Vector). Images were collected with an LSM 510 confocal laser scanning microscope (Carl Zeiss) with 63 × /1.4 oil objective. The frame size was 1,024 × 1,024. The manufacturer's software was used for data acquisition and ImageJ for fluorescence profiles. The weighted colocalization coefficients were calculated using AIM (Carl Zeiss).

### *In vivo* Delivery of miRNAs and Measurement of Plasma LDL Cholesterol

All animal experiments were approved by the Institutional Animal Care and Use Committee of New York University Grossman School of Medicine. *Ldlr*^−/−^ mice were obtained from Jackson Laboratories and bred to C57BL/6 mice to generate *Ldlr*^+/−^ mice. Mice were housed in cages at 21 ± 2°C with a 12-h dark/light cycle and allowed access to chow diet and water *ad libitum*. Lipid nanoparticles (LNPs) containing control miRNA or miR-224 were provided by Regulus Therapeutics and administered to 8- to 12-week-old male *Ldlr*^+/−^ mice by intravenous injection at 2 mg/kg twice weekly for 4 weeks. As a control, *Ldlr*^+/−^ mice were injected with a similar volume of PBS by intravenous injection twice weekly for 4 weeks. At sacrifice, mice were anesthetized with isoflurane, exsanguinated by cardiac puncture, and perfused with PBS. Plasma was separated by centrifugation, and LDL cholesterol concentrations were measured using an enzymatic colorimetric assay (Wako Diagnostics) according to the manufacturer's protocol. Livers were snap-frozen under liquid nitrogen and stored at −80°C for RNA and protein analysis.

### Statistical Analysis

Data are presented as mean ± the standard error of the mean (SEM) (*n* is noted in the figure legends), and the statistical significance of differences was evaluated with an unpaired two-sided Student *t*-test. Significance was accepted at the level of *P* < 0.05. Data analysis was performed using GraphPad Prism software (GraphPad).

## Results

### *PCSK9, IDOL*, and *HMGCR* Are *Bona Fide* Targets of miR-224 and miR-520d

**Figure 1:**
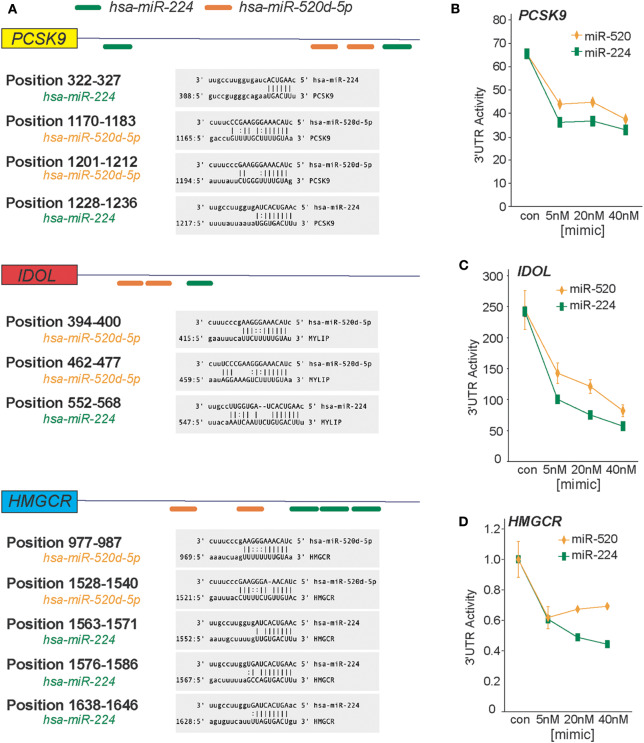
miR-224 and miR-520d target the 3′-UTRs of *PCSK9, IDOL*, and *HMGCR*. **(A)** *In silico* prediction of putative miRNA response elements for miR-224 (green) and miR-520d (orange) in the 3′-UTR of human *PCSK9, IDOL*, and *HMGCR*. Positional details of microRNA response elements are depicted in gray boxes. Predictions were based on the miRanda algorithm (http://www.microRNA.org). **(B–D)** Relative activity of *PCSK9* 3′-UTR luciferase reporter **(B)**, *IDOL* 3′-UTR luciferase reporter **(C)**, and *HMGCR* 3′-UTR luciferase reporter **(D)** constructs transfected in HEK-293T cells treated with increasing concentrations of miR-224, miR-520d, or control miR mimic.

**Figure 2:**
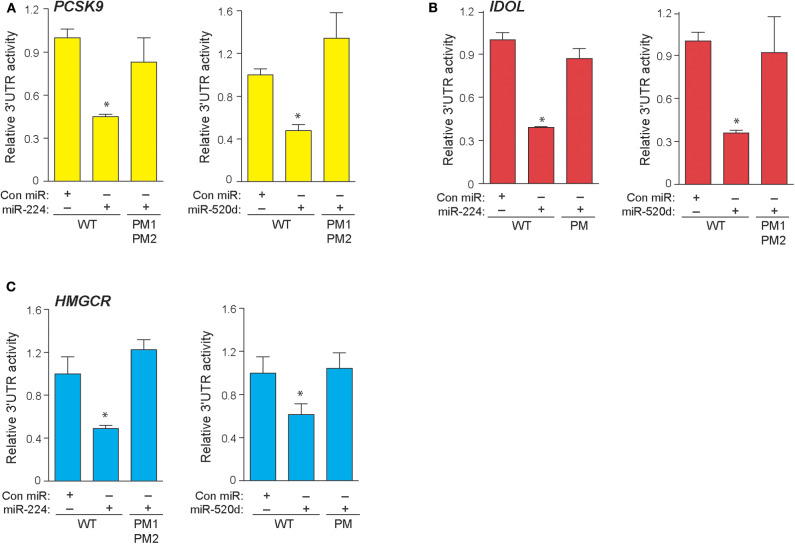
Point mutations in 3′-UTRs of *PCSK9, IDOL*, and *HMGCR* depress effects of miR-224 and miR-520d. **(A–C)** Relative activity of *PCSK9* 3′-UTR luciferase reporter **(A)**, *IDOL* 3′-UTR luciferase reporter **(B)**, and *HMGCR* 3′-UTR luciferase reporter **(C)** constructs with or without point mutations (PM) transfected in HEK-293T cells treated miR-224, miR-520d, or control miR mimic. Data are the mean ± SEM of triplicate samples from a single experiment and are representative of three independent experiments. *P*-values were calculated using two-tailed Student's *t*-test. *P < 0.05.

In a genome-wide screen of miRNAs altered by cellular cholesterol content (16), we found that miR-520d was upregulated 3.57-fold in cholesterol-depleted macrophages compared to cholesterol-loaded macrophages (Supplementary Figure 1). To gain insight into the function of miR-520d, we analyzed its potential gene targets using miRNA target prediction algorithms (e.g., TargetScan and miRanda) and identified multiple putative binding sites for miR-520d in the 3′-UTR of genes that modulate LDLR abundance, including the LDLR chaperone proteins PCSK9 and IDOL and the rate-limiting enzyme in cholesterol biosynthesis HMGCR (Figure 1A). During our analysis, we noted multiple additional sites for a second miRNA, miR-224, in the 3′-UTRs of these same genes (Figure 1A). To directly assess the effects of miR-224 and miR-520d on *PCSK9, IDOL*, and *HMGCR* expression, the 3′-UTRs of these human mRNAs were fused to a luciferase reporter plasmid. Using miRNA mimics, we found that miR-224 and miR-520d dose-dependently reduced the activity of the 3′-UTR of *PCSK9, IDOL*, and *HMGCR* by 50–80% compared to a control miRNA mimic (Figures 1B–**D**). Notably, this regulation by miR-224 and miR-520d was specific, as point mutations introduced in the predicted miRNA response elements in the 3′-UTR of *PCSK9, IDOL*, and *HMGCR* (Figures 2A–**C**) relieved repression by miR-224 and miR-520d and restored 3′-UTR activity to control levels.

**Figure 3:**
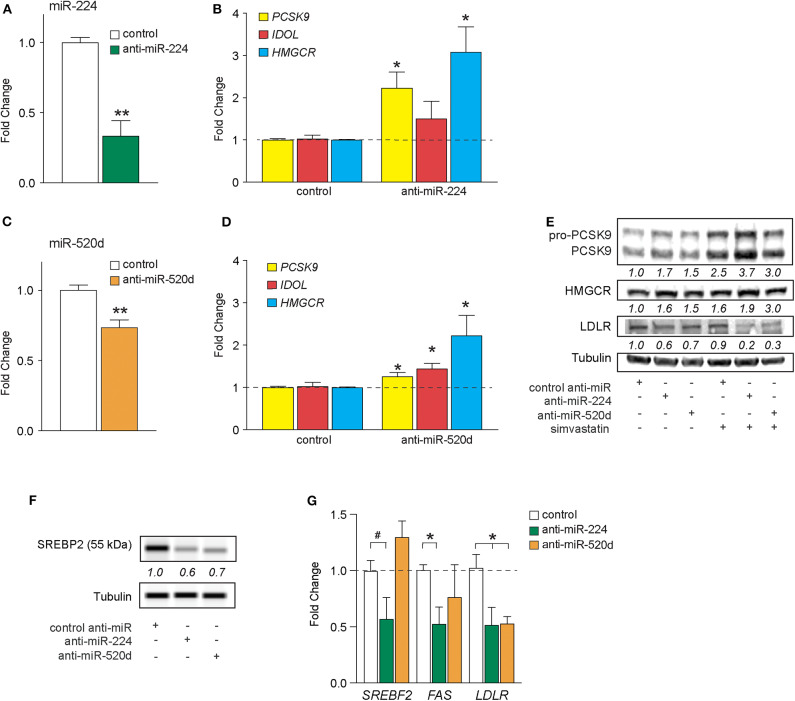
Inhibition of miR-224 and miR-520d regulates mRNA expression of *PCSK9, IDOL*, and *HMGCR* in human hepatocytes. **(A,B)** qRT-PCR analysis of miR*-*224 **(A)** and *PCSK9, IDOL*, or *HMGCR* **(B)** expression levels in HepG2 cells transfected with an inhibitor of miR-224 or a control inhibitor. **(C,D)** qRT-PCR analysis of miR*-*520d **(C)** and *PCSK9, IDOL*, or *HMGCR* **(D)** expression levels in HepG2 cells transfected with an inhibitor of miR-520d or a control inhibitor. **(E)** Western blot analysis of PCSK9, HMGCR, LDLR, and tubulin (internal control) in HepG2 cells transfected with anti-miR-224, anti-miR-520d, or control anti-miR and left untreated or treated with simvastatin (5 μM). Relative quantification is shown below the bands. **(F)** Representative western blot analysis of SREBP2 in HepG2 cells transfected with anti-miR-224, anti-miR-520d, or control anti-miR. Quantification of two independent experiments shown below bands. **(G)** qRT-PCR analysis of *SREBF2, FAS*, and *LDLR* in HepG2 cells transfected with anti-miR-224, anti-miR-520d, or control anti-miR. Data are the mean ± SEM of three independent experiments. *P*-values were calculated using two-tailed Student's *t*-test. ^#^*P* < 0.1, *P < 0.05, **P < 0.01.

*In silico* analysis of deposited high-throughput sequencing and crosslinking immunoprecipitation (HiTS-CLIP) experiments of human Argonaute (AGO) in hepatocellular carcinoma cells [GSE64680 (31)] confirmed AGO2 occupancy at predicted miR-224 and miR-520d response elements in the 3′-UTRs of *PCSK9, IDOL*, and *HMGCR* (Supplementary Figures 2–4). To understand the effects of endogenous miR-224 and miR-520d on these target mRNAs in hepatic cells, we transfected HepG2 cells with antisense oligonucleotide inhibitors of miR-224 and miR-520d or a control oligonucleotide. Compared to a control inhibitor, anti-miR-224 reduced levels of miR-224 in HepG2 cells by 70%, indicating efficient inhibition of endogenous miR-224 (Figure 3A). Correspondingly, we found that anti-miR-224 increased levels of *PCSK9* and *HMGCR* mRNAs by 2-fold to 3-fold and of *IDOL* mRNA to a lesser extent (Figure 3B). Similarly, anti-miR-520d reduced endogenous levels of miR-520d (Figure 3C) and increased levels of *PCSK9* and *IDOL* mRNAs by 30–40% and of *HMGCR* mRNA by 200% (Figure 3D). Furthermore, anti-miR-224 and anti-miR-520d increased protein levels of PCSK9 and HMGCR in HepG2 cells under basal conditions and after treatment with simvastatin (Figure 3E, Supplementary Figure 7), which was accompanied by a marked reduction in LDLR protein levels (Figure 3E, Supplementary Figure 7). As increased levels of HMGCR would be expected to increase cholesterol synthesis, we next examined whether anti-miR-224 and anti-miR-520d altered the SREBP2 pathway. SREBP2 levels in the nucleus are regulated by cellular cholesterol content. Under low-sterol conditions, SREBP2 is released from the endoplasmic reticulum and undergoes cleavage to its mature form that traffics to the nucleus to activate gene transcription, whereas under cholesterol-replete conditions, SREBP2 processing is inhibited. Consistent with the increase in HMGCR, we observed a decrease in mature SREBP2 protein in HEPG2 cells treated with anti-miR-224 and anti-miR-520d compared to control anti-miR, as measured by western blotting (Figure 3F, Supplementary Figure 7). The decrease in SREBP2 in anti-miR-224-treated cells corresponded with a decrease in mRNA levels of genes containing SREBP response elements (SREs) in their promoters, including *SREBF2* itself, *FAS*, and *LDLR* (Figure 3G). However, in anti-miR-520d-treated HEPG2 cells, we only observed a significant decrease in *LDLR* mRNA levels (Figure 3G), which may reflect its less potent effects on its target genes, and by extension, SREBP2 levels. Notably, we observed no change in expression of miRNAs known to directly target *LDLR*, such as miR-27b and miR-128, in HEPG2 cells treated with anti-miR-224 and anti-miR-520d (Supplementary Figure 5). Collectively, these data identify *PCSK9, IDOL*, and *HMGCR* as targets of miR-224 and miR-520d and indicate that inhibition of miR-244 and miR-520d could be used to regulate hepatic expression of LDLR at both the mRNA and protein levels.

### miR-224 and miR-520d Modulate LDLR Expression in Hepatic Cells Through Their Actions on *PCSK9, IDOL*, and *HMGCR*

**Figure 4:**
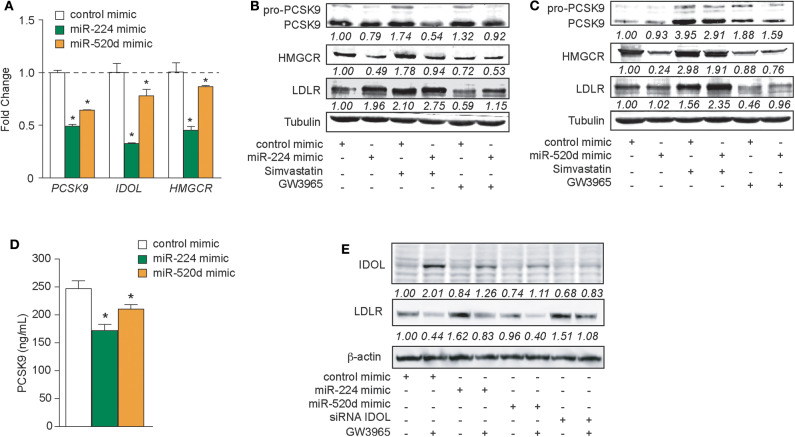
Increased expressions of miR-224 and miR-520d regulate PCSK9, HMGCR, IDOL, and LDLR in human hepatocytes. **(A)** qRT-PCR analysis of *PCSK9, IDOL*, and *HMGCR* in HepG2 cells transfected with miR-224, miR-520d, or control miR mimic. **(B,C)** Western blot analysis of PCSK9, HMGCR, and LDLR in HepG2 cells transfected with miR-224 **(B)**, miR-520d **(C)**, or control miR mimic and subsequently treated with simvastatin (5 μM) or GW3965 (1 μM). Relative quantification is shown below the band blots. **(D)** PCSK9 concentration measured with ELISA in supernatants derived from HepG2 cells transfected with miR-224, miR-520d, or control miR mimic. **(E)** Western blot analysis of IDOL and LDLR in HepG2 cells transfected with miR-224, miR-520d, or control miR mimic or a siRNA targeting IDOL and subsequently treated with GW3965 (1 μM). Relative quantification is shown below the band blots. Data are the mean ± SEM of triplicate samples from a single experiment and are representative of three independent experiments **(A,D)**. *P*-values were calculated using two-tailed Student's *t*-test. *P < 0.05.

To test whether delivery of miR-224 or miR-520d could be used to regulate hepatic expression of LDLR accessory proteins that control LDLR cell surface expression and, thus, LDL clearance, we transfected HepG2 cells with miR-224 or miR-520d or control miRNA mimics and measured the expression of their target genes at the mRNA and protein levels. Compared to control mimics, miR-224 potently inhibited *PCSK9, IDOL*, and *HMGCR* mRNA levels as measured by qRT-PCR (Figure 4A). By comparison, miR-520d reduced *PCSK9, IDOL*, and *HMGCR* mRNA by only 10–40% (Figure 4A), suggesting that miR-520d has more modest effects on its mRNA targets. Furthermore, miR-224 strongly reduced protein levels of PCSK9 and HMGCR in HepG2 cells under basal conditions and after treatment with simvastatin, which upregulates PCSK9 and HMGCR mRNA (Figure 4B, Supplementary Figure 6). Notably, miR-224 repression of PCSK9 and HMGCR was associated with a doubling in LDLR protein expression in unstimulated HepG2 cells and a 30% increase in LDLR protein levels in simvastatin-treated cells (Figure 4B, Supplementary Figure 6). Similar to miR-224, miR-520d reduced PCSK9 and HMGCR protein levels in HepG2 cells treated with simvastatin; however, its effects in untreated cells were modest (Figure 4C, Supplementary Figure 8). Simvastatin-treated HepG2 cells transfected with miR-520d showed a 50% increase in LDLR protein expression compared to a control miRNA (Figure 4C, Supplementary Figure 8). Together, these data indicate that delivery of miR-224 or miR-520d mimics can be used to increase LDLR protein expression in hepatic cells in the setting of statin treatment.

As PCSK9 can be secreted by the liver, we next measured the effects of miR-224 or miR-520d mimics on PCSK9 secretion by HepG2 cells. We treated HepG2 cells with control, miR-224, or miR-520d mimics and collected cell culture supernatants for analysis. Compared to a control miRNA, miR-224 reduced PCSK9 concentrations in the media by 30%, as measured by ELISA (Figure 4D). Similarly, compared to control, miR-520d reduced PCSK9 secretion by HepG2 cells by 20% (Figure 4D). Together, these data indicate that miR-224 and miR-520d efficiently reduce both intracellular and secreted PCSK9 levels in hepatic cells.

The LDLR chaperone protein IDOL is rapidly degraded by the proteasome in unstimulated cells but can be induced by activation of the LXR nuclear hormone receptor (9). Thus, to investigate the effects of miR-224 and miR-520d on IDOL protein expression, HepG2 cells were treated with the proteasome inhibitor MG132 and transfected with miR-224 or miR-520d mimics in the presence and absence of the LXR agonist GW3965. As shown in Figure 4E, Supplementary Figure 9, HepG2 cells treated with control miRNA mimics show low levels of IDOL protein, and IDOL protein is increased ~2-fold by GW3965. As expected, the GW3965-mediated increase in IDOL protein was associated with a 60% reduction of LDLR protein levels (Figure 4E, Supplementary Figure 9). Transfection of HepG2 cells with miR-224 mimics reduced basal and GW3965-induced IDOL protein levels, by 15 and 75%, respectively, compared to control mimic. Notably, under basal conditions, miR-224 mimics increased LDLR levels to an extent comparable to that seen after transfection with an *IDOL* siRNA (Figure 4E, Supplementary Figure 9). Interestingly, transfection of HepG2 cells with miR-520d mimics also reduced IDOL protein levels under basal and GW3965-treated conditions but did not significantly change levels of LDLR protein (Figure 4E, Supplementary Figure 9). Together, these data indicate that miR-224 and miR-520d can reduce levels of IDOL protein under basal conditions and upon LXR activation.

### miR-224 and miR-520d Regulate Surface Expression of LDLR and LDL Clearance

**Figure 5:**
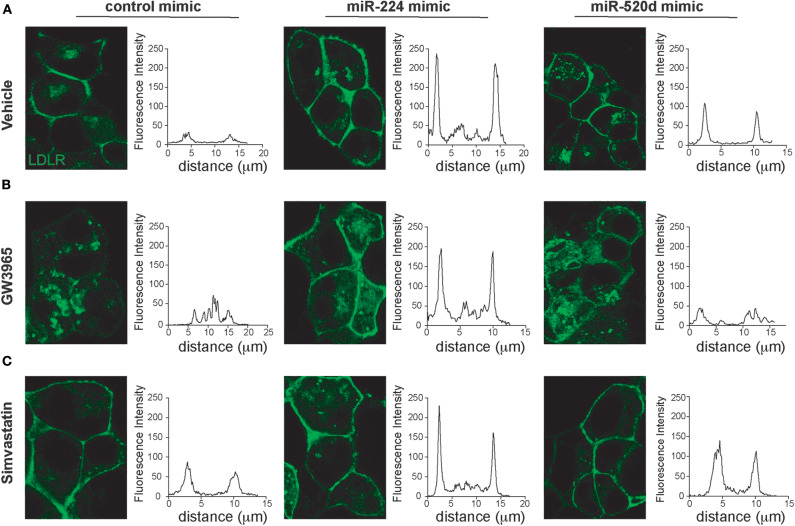
miR-224 and miR-520d control cell surface expressions of LDLR. **(A–C)** Representative images of HepG2 cells stably expressing LDLR-GFP (green) transfected with miR-224, miR-520d, or a control miRNA mimic and treated with a control vehicle **(A)**, GW3965 (**B**, 1 μM), or simvastatin (**C**, 5 μM) or a vehicle control. Graphs to the right of each of the images depict the mean fluorescence intensity across a line drawn through a single cell. Data are representative of three independent experiments.

Our data suggest that miR-224 and miR-520d can regulate LDLR protein levels by targeting accessory proteins that regulate its degradation. To understand how miR-224 and miR-520d affect cell surface expression of LDLR, and thus LDL binding, we used HepG2 cells stably expressing a GFP-tagged LDLR (HepG2-LDLR-GFP). The LDLR-GFP transgene does not contain the native LDLR 3′-UTR, and thus, this cell line is ideal to investigate how miR-224 or miR-520d regulation of chaperone proteins (e.g., PCSK9 and IDOL) affects LDLR cell surface expression, without potential direct effects of the miRNAs on the native LDLR mRNA. As shown in Figure 5A, HepG2-LDLR-GFP cells transfected with miR-224 or miR-520d mimics under basal conditions show increased mean cell surface expression of LDLR-GFP when compared to control mimics. To attempt to discriminate the effects of miR-224 and miR-520d on PCSK9- and IDOL-mediated LDLR regulation, we took advantage of their differential regulation by statins and LXR activators. In control-mimic-treated HepG2-LDLR-GFP cells stimulated with the LXR agonist GW3965, which increases expression of *IDOL*, we observed internalization of the LDLR (Figure 5B, left panel). Transfection with miR-224 restored LDLR-GFP cell surface expression in GW3965-treated cells (Figure 5B, middle panel), in agreement with its ability to reduce cellular IDOL protein expression (Figure 4E, Supplementary Figure 9). By contrast, transfection with miR-520d only partially restored LDLR-GFP cell surface expression (Figure 5B, right panel), consistent with the differential effects of miR-520d and miR-224 on total LDLR protein levels (Figure 4E, Supplementary Figure 9). Finally, we examined the effects of miR-224 and miR-520d in HepG2-LDLR-GFP cells treated with simvastatin, a condition that increases expression of PCSK9. As observed in vehicle-treated cells, miR-224 markedly increased mean cell surface expression of LDLR-GFP in simvastatin-treated cells when compared to control mimic, suggesting that it effectively boosts cell surface expression of LDLR levels under conditions that increase PCSK9 protein (Figure 5C). Similarly, miR-520d increased cell surface expression of LDLR-GFP in simvastatin-treated cells when compared to control mimic, although to a lesser extent than miR-224 (Figure 5C).

**Figure 6:**
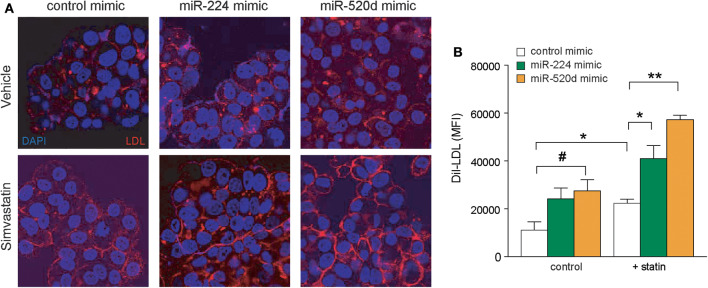
Enhanced expressions of miR-224 and miR-520d increase diI-LDL binding to LDLR. **(A)** Representative microscopic images of diI-LDL (red) in HepG2 cells transfected with miR-224, miR-520d, or control miRNA mimic and subsequently treated with control vehicle or simvastatin (5 μM). Counterstaining with DAPI to visualize DNA (blue) in the nucleus. **(B)** Quantification of images of diI-LDL (red) in HepG2 cells transfected with miR-224, miR-520d, or control miRNA mimic and subsequently treated with control vehicle or simvastatin (5 μM). *P*-values were calculated using two-tailed Student's *t*-test. ^#^*P* < 0.1, *P < 0.05, **P < 0.01.

To investigate the effects of miR-224 and miR-520d on LDLR function, we next measured the binding of fluorescently labeled LDL (diI-LDL) to native HepG2 cells transfected with control, miR-224, or miR-520d mimics. Under basal conditions, transfection of HepG2 cells with miR-224 or miR-520d mimics increased diI-LDL binding ~2-fold compared to control miR mimics, similar to the effects of statin treatment, although only treatment with miR-520d reached statistical significance (Figures 6A,B). Notably, in statin-treated HepG2 cells, miR-224 and miR-520d mimics further augmented diI-LDL binding by 2-fold to 3-fold (Figures 6A,B). Collectively, our data suggest that miR-224 and miR-520d may be effective in increasing LDLR function in concert with statin treatment by repressing expression of accessory proteins that reduce LDLR cell surface expression.

### Therapeutic Delivery of miR-224 Decreases LDL Cholesterol *in vivo*

**Figure 7:**
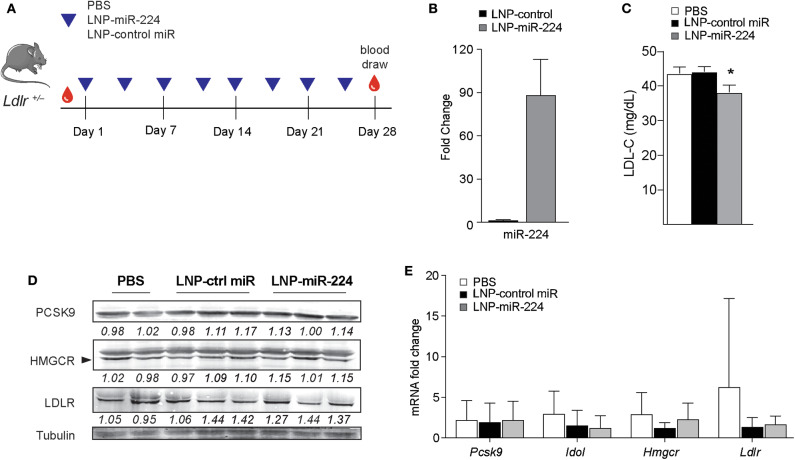
miR-224 reduces plasma levels of LDL cholesterol *in vivo*. **(A)** Experimental design to test the effects of miR-224 overexpression *in vivo*. *Ldlr*^+/−^ mice (*n* = 7–8) were intravenously injected with PBS or 2 mg/kg of lipid nanoparticles (LNPs) containing miR-224 or control miRNA mimic 2×/week for 4 weeks. **(B)** qRT-PCR analysis of miR-224 in livers of treated *Ldlr*^+/−^ mice. **(C)** Levels of LDL cholesterol (LDL-C) in plasma of treated *Ldlr*^+/−^ mice at the end of the study. **(D)** Western blot analysis of PCSK9, HMGCR, and LDLR proteins in livers of treated *Ldlr*^+/−^ mice. Relative quantification is shown below the blots. **(E)** Quantitative PCR analysis of *PCSK9, IDOL, HMGCR*, and *LDLR* mRNA levels in livers of treated *Ldlr*^+/−^ mice. Data are the mean ± SEM of *n* = 7–8 mice per group. *P*-values were calculated using a one-way ANOVA. *P < 0.05.

To understand the effects of miR-224 *in vivo*, we overexpressed miR-224 in the livers of *Ldlr*^+/−^ mice using LNP-mediated delivery. While C57BL/6 mice carry the majority of cholesterol in HDL, *Ldlr*^+/−^ mice have a humanized lipoprotein profile (Supplementary Figure 5B), allowing us to assess effects of miR-224 on plasma levels of LDL cholesterol. We injected *Ldlr*^+/−^ mice twice weekly with LNP-miR-224 or LNP-control miRNA or PBS intravenously for 4 weeks and fed the mice a chow diet (Figure 7A). We observed no significant changes in body weight between the treatment groups (data not shown). After LNP-miR-224 treatment, hepatic levels of miR-224 were markedly increased as compared to LNP-control miRNA treatment, confirming efficient delivery of miR-224 to the liver (Figure 7B). Consistent with this, we observed a 15% reduction in plasma LDL cholesterol levels in *Ldlr*^+/−^ mice treated with LNP-miR-224, compared to treatment with LNP-control miRNA or PBS (Figure 7C). Despite these changes, we did not detect significant differences in the expression of the miR-224 target genes PCSK9, IDOL and HMGCR, or LDLR, in the livers of LNP-miR-224-treated mice, compared to LNP-control miRNA- or PBS-treated mice (Figures 7D,E, Supplementary Figures 5C, 10). While the reasons for this are unknown, it may be due to the high variability in expression of these genes that we observed among treated mice. These data suggest that miR-224 overexpression *in vivo* lowers plasma levels of LDL cholesterol, but further studies will be needed to better understand its mechanisms of action.

## Discussion

High plasma LDL cholesterol levels are a major risk factor for atherosclerosis, the underlying cause of coronary artery disease (CAD) (32). CAD remains the number one cause of morbidity and mortality in the United States (32), and new therapies are urgently needed to reduce the burden of disease. While much research has focused on the transcriptional regulation of cholesterol metabolism, recent studies from our lab and others have highlighted the impact of miRNAs on posttranscriptional control of the cholesterol regulatory circuitry. A number of studies have established roles for miRNAs in regulating cellular cholesterol efflux and HDL biogenesis [e.g., miR-33 (15–17, 33), miR-144 (21, 23), and miR-223 (26)], nuclear SREBP accumulation and lipid synthesis [e.g., the miR-96/miR-182/miR-183 cluster (34)], and assembly of apoB-containing lipoproteins [e.g., miR-30c (35)]. In addition, several miRNAs have been shown to directly target the 3′-UTR of the LDLR and to repress LDLR protein expression, including miR-128, miR-148a, miR-130b, and miR-301b (29, 30). Our study adds to the growing understanding of miRNA regulation of cholesterol homeostasis by identifying two miRNAs that regulate gene networks that impact hepatic LDLR expression and LDL binding. We show that miR-224 and miR-520d can regulate three arms that control LDLR cell surface expression: the rate-limiting enzyme for cholesterol synthesis HMGCR, the chaperone protein PCSK9 that regulates LDLR recycling and lysosomal degradation, and the E3-ubiquitin ligase IDOL that regulates LDLR ubiquitination and proteasomal degradation. Using gain- and loss-of-function studies, we confirmed *PCSK9, IDOL*, and *HMGCR* as targets of miR-224 or miR-520d and demonstrated that overexpression of these miRNAs can be used as a strategy to upregulate LDLR expression and function in hepatocytes. Notably, the effects of miR-224 or miR-520d on hepatic LDLR cell surface expression and LDL binding were additive to the effects of statins, currently the standard of care to lower plasma levels of LDL cholesterol (36), suggesting that such an approach could be used in concert with statin therapy to increase LDLR function.

The statin class of drugs inhibits the rate-limiting enzyme in cholesterol synthesis HMGCR, leading to the feedback activation of SREBP, a transcription factor that drives LDLR expression (36). This upregulates hepatic LDLR expression and increases the clearance of circulating LDL, effectively lowering its plasma concentrations. However, one reason that statins are not more effective is that their induction of SREBP also drives expression of PCSK9, which removes the LDLR from the cell surface, thereby lessening its ability to clear LDL particles from the circulation. We find that both miR-224 and miR-520d simultaneously repress HMGCR and PCSK9, identifying a mechanism for overcoming this limitation. Importantly, we show that both miR-224 and miR-520d reduced secretion of PCSK9 to the extracellular environment, where it binds the LDLR for internalization and lysosomal degradation. In addition, the repression of LDLR degradation by miR-224 and miR-520d is reinforced by their targeting of the other known chaperone protein that targets the LDLR for degradation, IDOL. Notably, miR-224 and miR-520d repression of PCSK9 and IDOL was effective under conditions that increased expression of these targets, such as statin or LXR agonist treatment, respectively, and resulted in increased hepatic LDLR cell surface expression and LDL binding.

The discovery that loss-of-function mutations in the PCSK9 gene caused lower-than-normal LDL cholesterol levels and decreases in CAD (6) led to the rapid clinical development of monoclonal antibodies targeting PCSK9. Currently, two PCSK9 binding monoclonal antibodies, alirocumab and evolocumab, are approved for use with and without statins and have allowed achievement of low levels of plasma LDL cholesterol in high-risk patient populations (8). Other modalities to inhibit PCSK9, including antisense oligonucleotides and short interfering RNAs, are being actively pursued (37). Although several miRNAs, including miR-191, miR-222, and miR-224, have been identified to regulate PCSK9 in human liver cells (38), none are currently in clinical development. PCSK9 repression by miR-224 was previously reported (38, 39); however, those studies did not examine effects of the miR-224 on PCSK9 secretion or LDLR expression and function, nor did they examine effects on other arms of LDL regulation as we have performed here. Interestingly, miR-224 has been reported to be commonly dysregulated in human cancers including glioma, lung adenocarcinoma, and hepatocellular carcinoma, as well as cervical, prostate, breast, and colorectal cancer (40), indicating that it is unlikely to be a candidate for development as a lipid-lowering therapy. Multiple additional targets of miR-224 have been identified that may promote tumor cell proliferation and metastasis, including the cyclin-dependent kinase p21, SMAD4, PPP2R1B, TRIB1, RKIP, API5, CDC42, PAK2, and CDH1 [reviewed in Chen et al. (40)]. By contrast, miR-520 family members have been linked to tumor suppression in breast and esophageal cancers (41, 42), suggesting that this miRNA may be a better candidate for future studies.

In contrast to PCSK9, relatively little is known about posttranscriptional mechanisms that regulate IDOL and proteasomal degradation of the LDLR. IDOL controls LDLR receptor stability independent of SREBP and PCSK9 and is transcriptionally induced by activation of the LXR nuclear receptors (9). The LXRs are activated in the setting of cholesterol excess and regulate the expression of genes involved in cholesterol uptake, transport, efflux, and excretion. Upon its induction by LXR, IDOL binds to the cytoplasmic tail of the LDLR and promotes its ubiquitination by the UBE2D1/E1 complex (43, 44) and delivery to the multivesicular body protein-sorting pathway (45, 46). In mice, targeted deletion of IDOL increases cellular LDLR protein levels and LDL uptake, and this increase is additive with the effect of statin treatment (45). Moreover, studies in non-human primates indicate that manipulation of IDOL using LXR agonists or IDOL siRNAs can regulate plasma levels of LDL cholesterol (47). To date, the role of miRNAs in regulating IDOL has been relatively unexplored. Posttranscriptional regulation of IDOL expression by miR-19b was reported in the setting of breast cancer, where it was shown to promote cell migration and metastasis (48). However, the effects of miR-19b on IDOL-dependent LDLR regulation or LDL uptake were not examined in that study. Thus, our identification of miR-224 and miR-520d as posttranscriptional repressors of IDOL are the first to explore how miRNA regulation of this pathway can be used to alter LDL metabolism.

miRNAs represent a new class of therapeutic targets, and delivery of oligonucleotide inhibitors and mimics is being tested in multiple disease indications. miRNA inhibitors are currently in clinical trials for the treatment of the hepatitis C virus [i.e., miR-122 (49, 50)], scar tissue formation [i.e., MRG-201 (Miragen) targeting miR-29], wound healing and heart failure (i.e., MRG-110 targeting miR-29), and various forms of cancer [i.e., MRG-106 targeting miR-155 and RGLS-5579 (Regulus) targeting miR-10b (51)]. These inhibitors are typically single-stranded oligonucleotides with modifications that render them resistant to degradation, and they can be delivered subcutaneously, intravenously, or locally for maximal effects. However, strategies to enhance miRNA expression or action remain more challenging due to the double-stranded nature of such miRNA mimics. Delivery of double-stranded miRNA mimics shares many of the limitations associated with siRNA delivery, including injection site reactions. Strategies to overcome these limitations, such as the packaging of miRNA mimics into lipid-based nanoparticles or liposomes, have improved delivery of miRNAs to some tissues such as the liver, a major site of regulation of cholesterol homeostasis. miRNAs such as miR-224 and miR-520d that can target multiple components of the LDLR control pathway (e.g., *PCSK9, IDOL*, and *HMGCR*) and that are effective under different conditions (e.g., statin treatment or LXR activation) are thus promising new therapeutic targets. The development of miRNA mimic delivery systems with improved tissue specificity will facilitate future studies of overexpression of such miRNAs *in vivo* to test effects on plasma LDL cholesterol levels and atherosclerosis.

## Data Availability Statement

The miRNA microarray dataset has been deposited in Gene Expression Omnibus (GEO, https://www.ncbi.nlm.nih.gov/geo/ and is available under GSE144751. Processed sequence datasets GSE64680 were downloaded from GEO-database and further analyzed using Integrative Genomics Viewer (https://igv.org/).

## Author Contributions

KM, KR, AS, and CS designed the study and prepared the manuscript, with input from all authors. AS, CS, ES, AW, MA, SO, and WS performed experiments and data analyses. KM and PT designed experiments and guided the interpretation of the results.

## Conflict of Interest

The authors declare that the research was conducted in the absence of any commercial or financial relationships that could be construed as a potential conflict of interest.

## Supplementary Material

Supplementary Figure 1 Relative microRNA expression in cholesterol loaded vs. depleted macrophages.

Supplementary Figure 2 Snapshots of Integrative Genomics Viewer used to analyze human AGO HiTS-CLIP data of the 3′-UTR of *PCSK9*, occupation of AGO at miR-224 and miR-520d response elements (red rectangles) in human hepatic cells with different conditions is shown in blue.

Supplementary Figure 3 Snapshots of Integrative Genomics Viewer used to analyze human AGO HiTS-CLIP data of the 3′-UTR of *IDOL*, occupation of AGO at miR-224 and miR-520d response elements (red rectangles) in human hepatic cells with different conditions is shown in blue.

Supplementary Figure 4 Snapshots of Integrative Genomics Viewer used to analyze human AGO HiTS-CLIP data of the 3′-UTR of *HMGCR*, occupation of AGO at miR-224 and miR-520d response elements (red rectangles) in human hepatic cells with different conditions is shown in blue.

Supplementary Figure 5 **(A)** qRT-PCR analysis of miR*-*27b and miR-128 in HepG2 cells transfected with anti-miR-224 or anti-miR-520d or control anti-miR. Data are the mean ± s.e.m. of 3 independent experiments. **(B)** Plasma lipoprotein profile of *Ldlr*^+/−^ mice analyzed by FPLC. **(C)** Western blot analysis of PCSK9, HMGCR, and LDLR protein in livers of treated *Ldlr*^+/−^ mice.

Supplementary Figure 6 Representative uncropped Western blot of PCKS9, HMCGR, LDLR, and Tubulin. This figure accompanies Figure 4B.

Supplementary Figure 7 Representative uncropped Western blot of PCKS9, HMCGR, SREBP2, and Tubulin. This figure accompanies Figures 3E,F.

Supplementary Figure 8 Representative uncropped Western blot of PCKS9, HMCGR, LDLR, and Tubulin. This figure accompanies Figure 4C.

Supplementary Figure 9 Representative uncropped Western blot of IDOL, LDLR, and -actin. This figure accompanies Figure 4E.

Supplementary Figure 10 Representative uncropped Western blot of PCKS9, HMCGR, LDLR, and Tubulin. This figure accompanies Figure 7D.

Supplementary Table 1 Primers used to introduce point mutations in the MRE of the 3′UTR of PCSK9, IDOL, and HMGCR.

Supplementary Table 2 qRT-PCR primers used in this study.
