# Supplementary material for: LDL Receptor Pathway Regulation by miR-224 and miR-520d
Source: Front Cardiovasc Med. 2020 May 22;7:81. doi: 10.3389/fcvm.2020.00081 (PMC7256473; doi:10.3389/fcvm.2020.00081)

Supplementary Table 1. Primers used to introduce point mutations in the MRE of the 3'UTR of PCSK9, IDOL and HMGCR

**Genes (human)**	**miRNA**	**Point mutations**
PCSK9	hsa-miR-224	MRE 1:<br>FW: 5'-GATGTCCGTGGGCAGAATTATTGAGCTCTTGTTC-3'<br>RV: 5'-GAACAAGAGCTCAATAATTCTGCCCACGGACATC-3'<br>MRE 2:<br>FW: 5'-GCCGATGTCCGTGGGCAGAAAGTCAATTATTGAGCTCTTGTTCCGT-3'<br>RV: 5'-ACGGAACAAGAGCTCAATAATTGACTTTCTGCCCACGGACATCGGC-3'
	hsa-miR-520	MRE 1:<br>FW: 5'-CTGTTTTGCCTTGAAGATATTTATTCTGGGTGCATTTTTATTAATATG-3'<br>RV: 5'-CATATTAATAAAAATGCACCCAGAATAAATATCTTCAAGGCAAAACAGG-3'<br>MRE 2:<br>FW: 5'-CTAGACCTGTTTTGCTAATCAATTGAAGAC<br>ATATTTATTCTGGGTAAAGATGCATTTTTATTAATATGGTGACTTTTTAAA-3'<br>RV: 5'-TTTAAAAAGTCACCATATTAATAAAAATGC<br>ATCTTTACCCAGAATAAATATCTTCAAGTTTGATTAGCAAAACAGGTCTAG-3'
IDOL	hsa-miR-224	MRE:<br>FW: 5'-GGAAGGCAAACAGGTTTACAAATCAATTGTCAGACTTTTAAA-3'<br>RV: 5'-TTTAAAAGTCTGACAATTGATTTGTAAACCTGTTTGCCTTCC-3'
	hsa-miR-520	MRE 1:<br>FW: 5'-ACTGGCAGCAGATAATGTTTGAATTACTTTTAAGAATTT<br>CATTCTTTTTGTATGGTCATGGAGCTCCAACCATTTT-3'<br>RV: 5'-AAAATGGTTGGAGCTCCATGACCATACAAAAAGAATGA<br>AATTCTTAAAAGTAATTCAAACATTATCTGCTGCCAGT -3'<br>MRE 2:<br>FW: 5'-GAGCTCCAACCATTTTTAATAGGAAAGTCTTAAGATAAT<br>TGTTGTCGTTTTAATGTCATTTCT-3'<br>RV: 5'-AGAAATGACATTAAAACGACAACAATTATCTTAAGACTT<br>TCCTATTAAAAATGGTTGGAGCTC-3'
HMGCR	hsa-miR-224	MRE 1/2:<br>FW: 5'-CGTAAAGCTTAAATTGCTTTTGTTTTTCTGAGCTTTTCATGGAAGTGG-3'<br>RV: 5'-CCACTTCCATGAAAAGCTCAGAAAAACAAAAGCAATTTAAGCTTTACG-3'<br>MRE 3:<br>FW: 5'- CCACTTCCATGAAAAGCTCAGAAAAACAAAAGCAATTTAAGCTTTACG -3'<br>RV: 5'- CCACTTCCATGAAAAGCTCAGAAAAACAAAAGCAATTTAAGCTTTACG -3'
	hsa-miR-520	MRE 1:<br>FW: 5'-CTTAAAAGACATTTTTATTTAGTTGAAAAATCTAGA-3'<br>RV: 5'-ACTGTATCAAATCTGTATATGTTGTAATAAAACT-3'<br>MRE 2:<br>FW: 5'-AGTTTTATTACAACATATACAGATTTGATACAGTTC-3'<br>RV: 5'-TAGATTTTTCAACTAAATAAAAATGTCTTTTAAG -3'

Supplementary Table 2. qRT-PCR primers used in this study

**Genes (human)**	**Forward Sequence**	**Reverse Sequence**
PCSK9	5’-AGGGGAGGACATCATTGGTG-3’	5’-CAGGTTGGGGGTCAGTACC-3’
IDOL	5’-CGAGGACTGCCTCAACCA-3’	5’-TGCAGTCCAAAATAGTCAACTTCT-3’
HMGCR	5’-GTCATTCCAGCCAAGGTTGT-3’	5’-GGGACCACTTGCTTCCATTA-3’
LDLR	5’-CTCTCTGCGAAGTCTGGACG-3’	5’-ACTAACCTGTCCGTACACCTTG-3’
GAPDH	5’GAAGGTGAAGGTCGGAGTC-3’	5’-GAAGATGGTGATGGGATTTC-3’

**Genes (mouse)**	**Forward Sequence**	**Reverse Sequence**
Pcsk9	5’-GAGACCCAGAGGCTACAGATT-3’	5’-AATGTACTCCACATGGGGCAA-3’
Idol	5’-AGGAGATCAACTCCACCTTCTG-3’	5’-ATCTGCAGACCGGACAGG-3’
Hmgcr	5’-TTGCAGAGCCGTCCTCTCTA-3’	5’-AGCAAGCTCCCATCACCAAG-3’
Ldlr	5’-CATGGCAGCCGGCAGTGTGA-3’	5’-TTGGGTCCCTCGCAGAGTGT-3’
Gapdh	5’TGTGAGGGAGATGCTCAGTG-3’	5’-TGTTCCTACCCCCAATGTGT-3’

Qiagen miScript Primer Assays	
hsa_miR-27b (MS00031668)	sequence not disclosed
hsa_miR-128 (MS00045850)	sequence not disclosed
hsa_miR-224 (MS00003878)	sequence not disclosed
hsa_miR-520d (MS00033866)	sequence not disclosed
RNU6 (MS00033740)	sequence not disclosed

Supplemental Figure 1

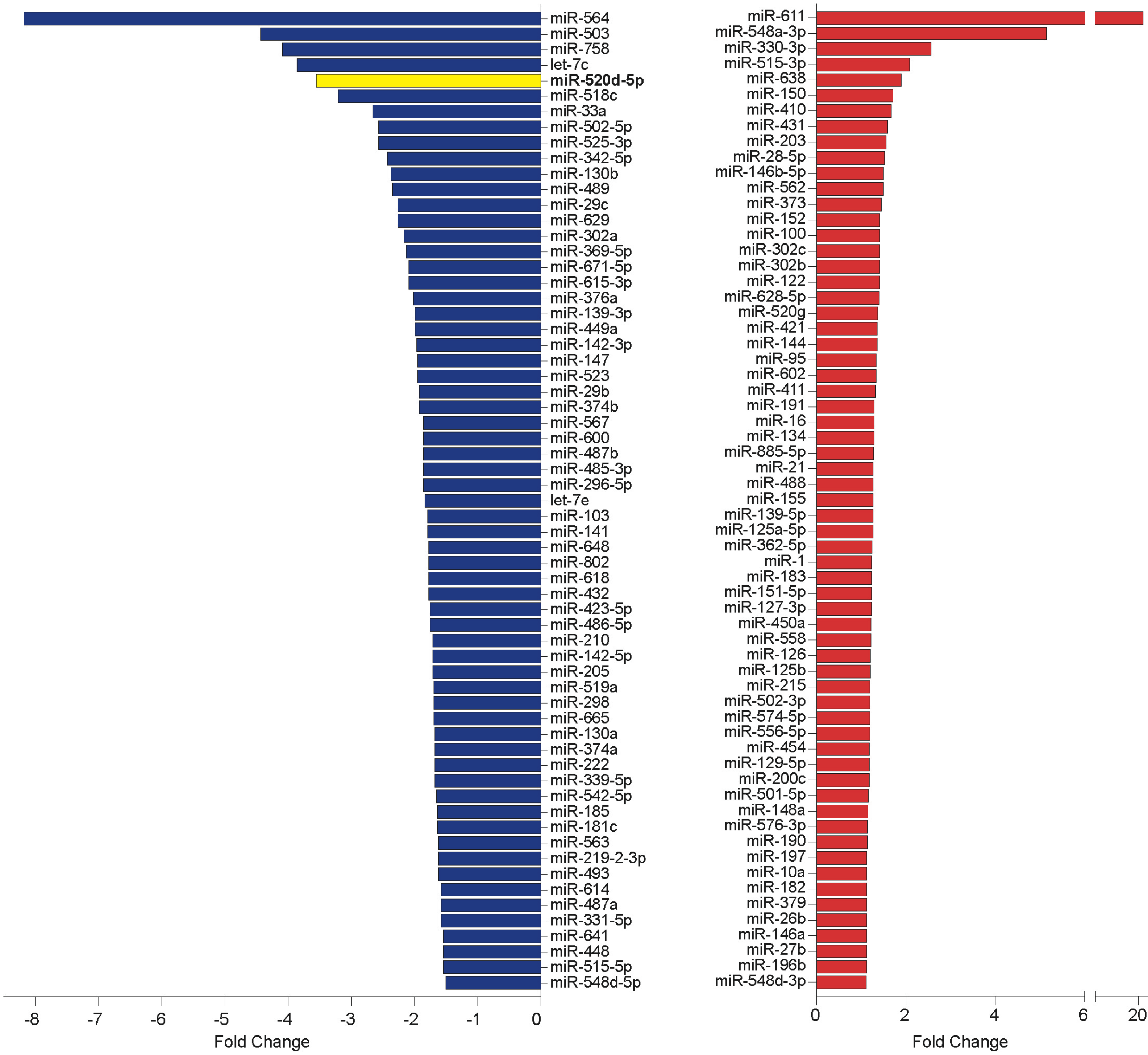

Supplemental Figure 2

PCSK9 3' UTR

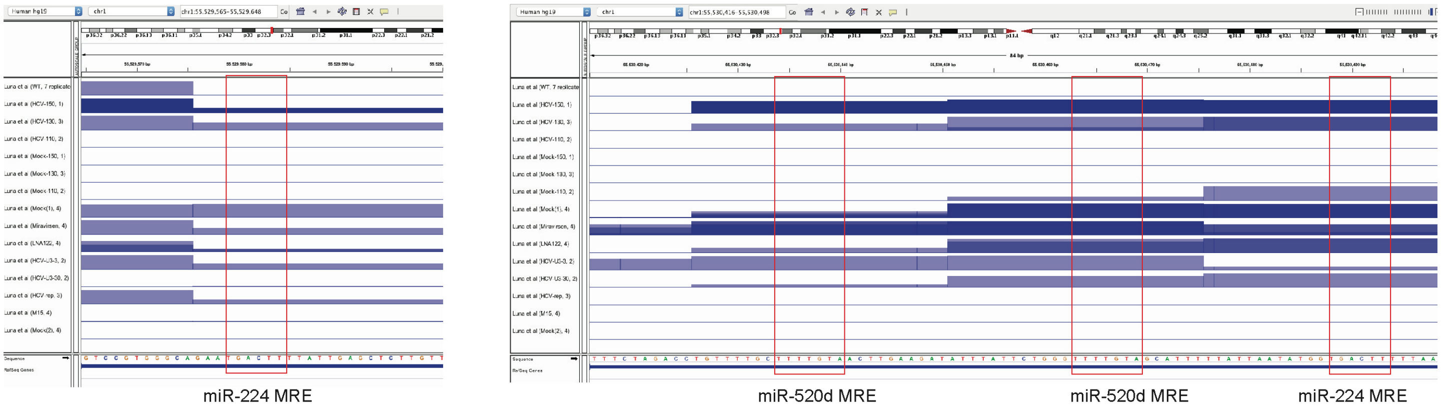

Supplemental Figure 3

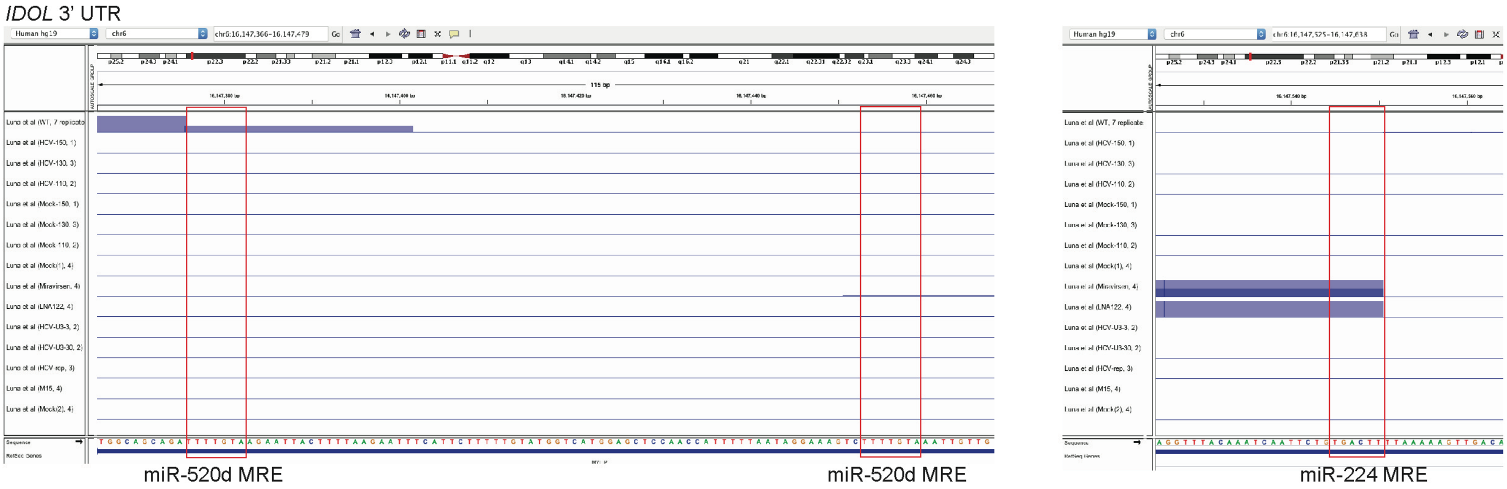

Supplemental Figure 4

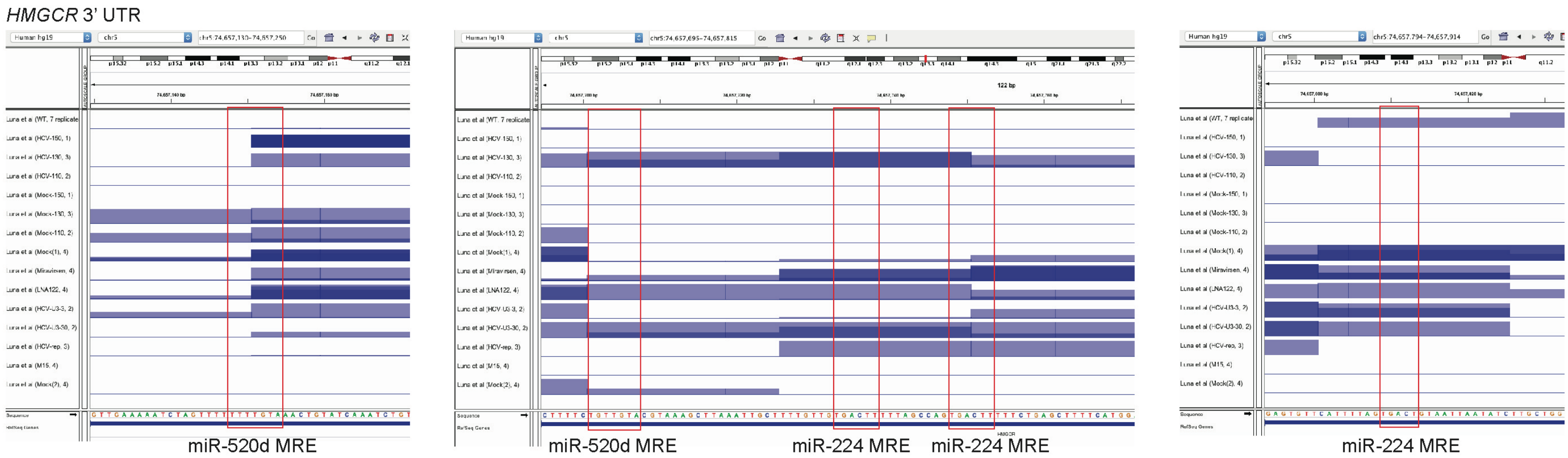

Supplemental Figure 5

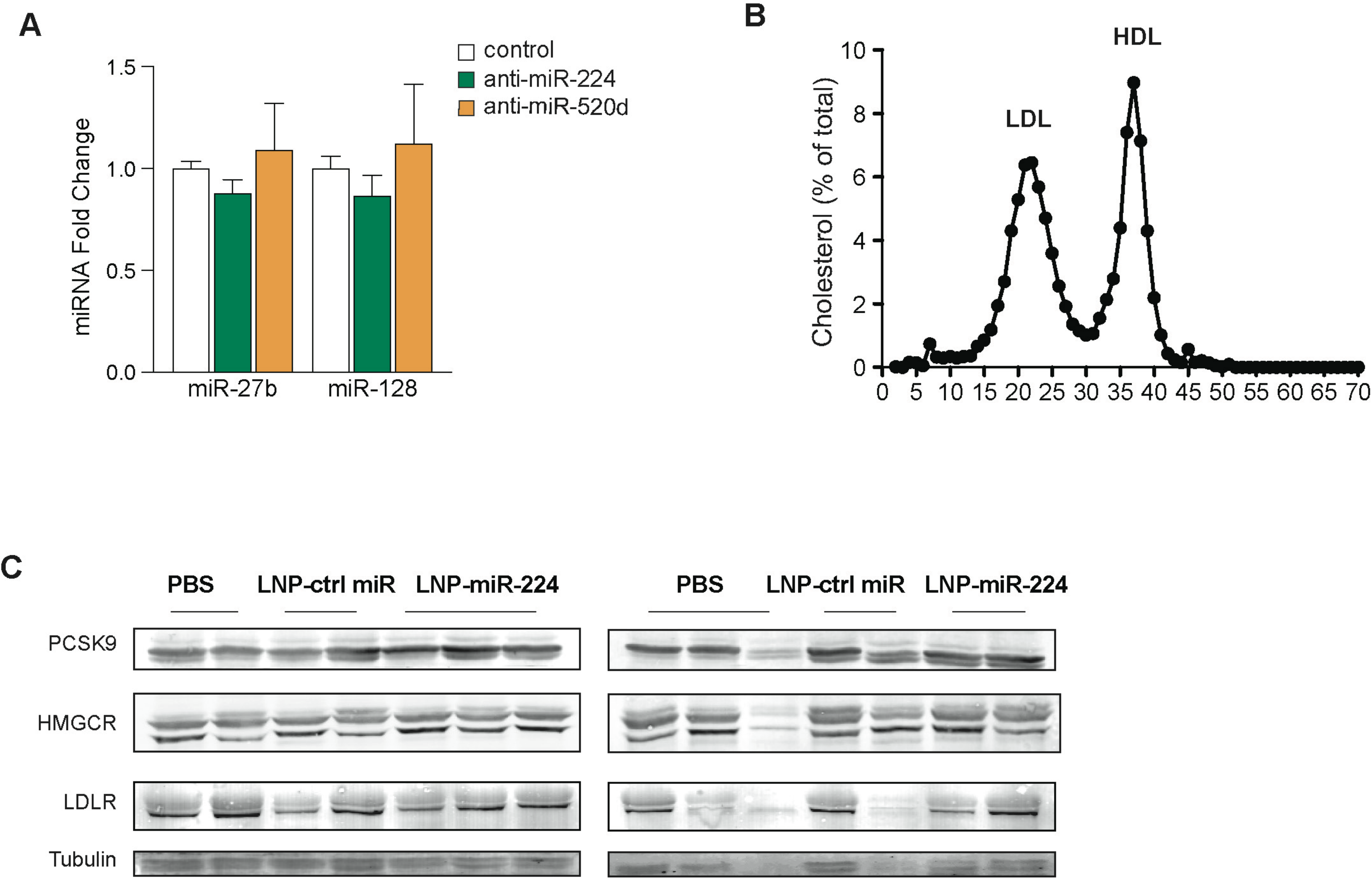

Supplemental Figure 6

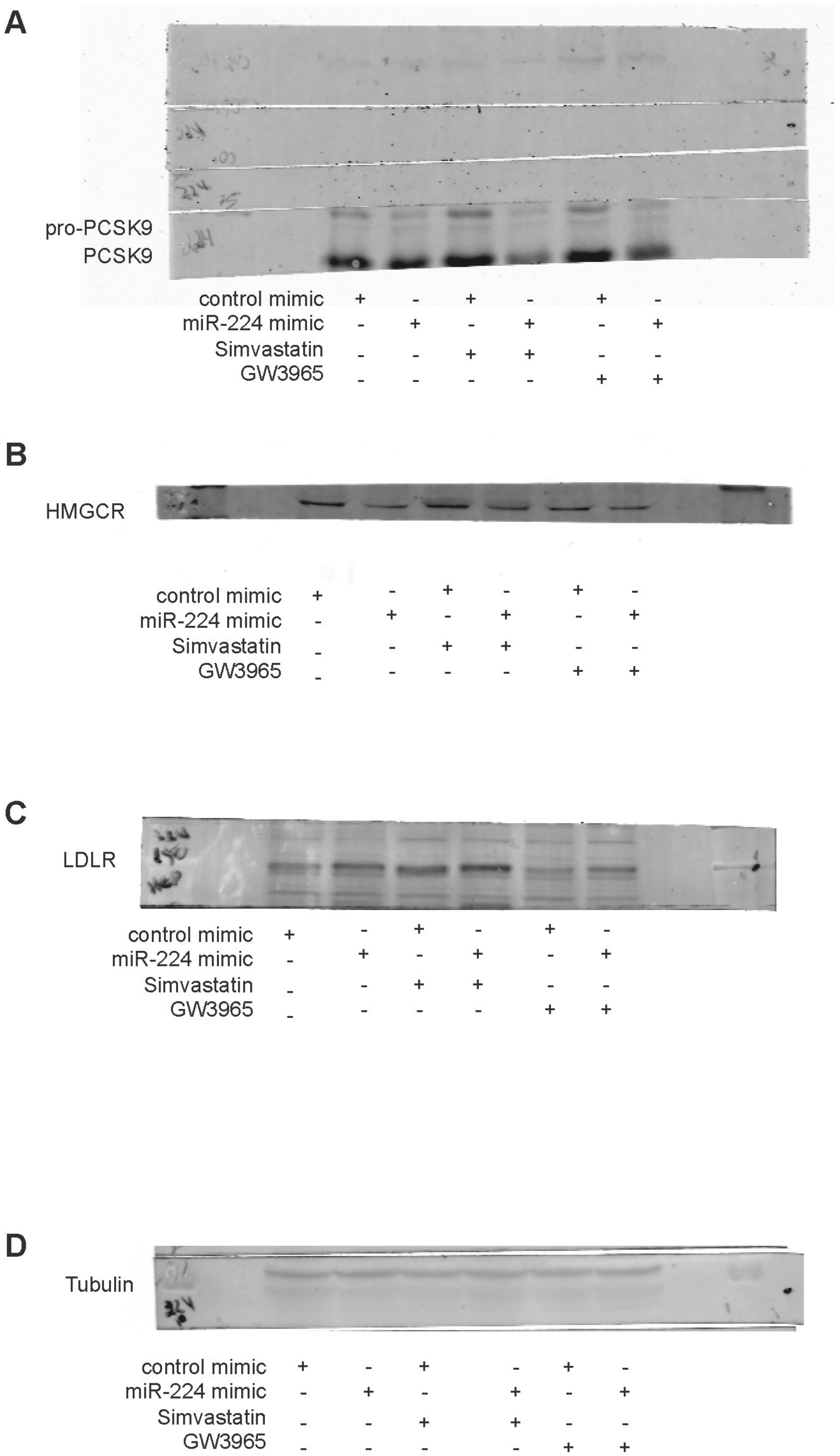

Supplemental Figure 7

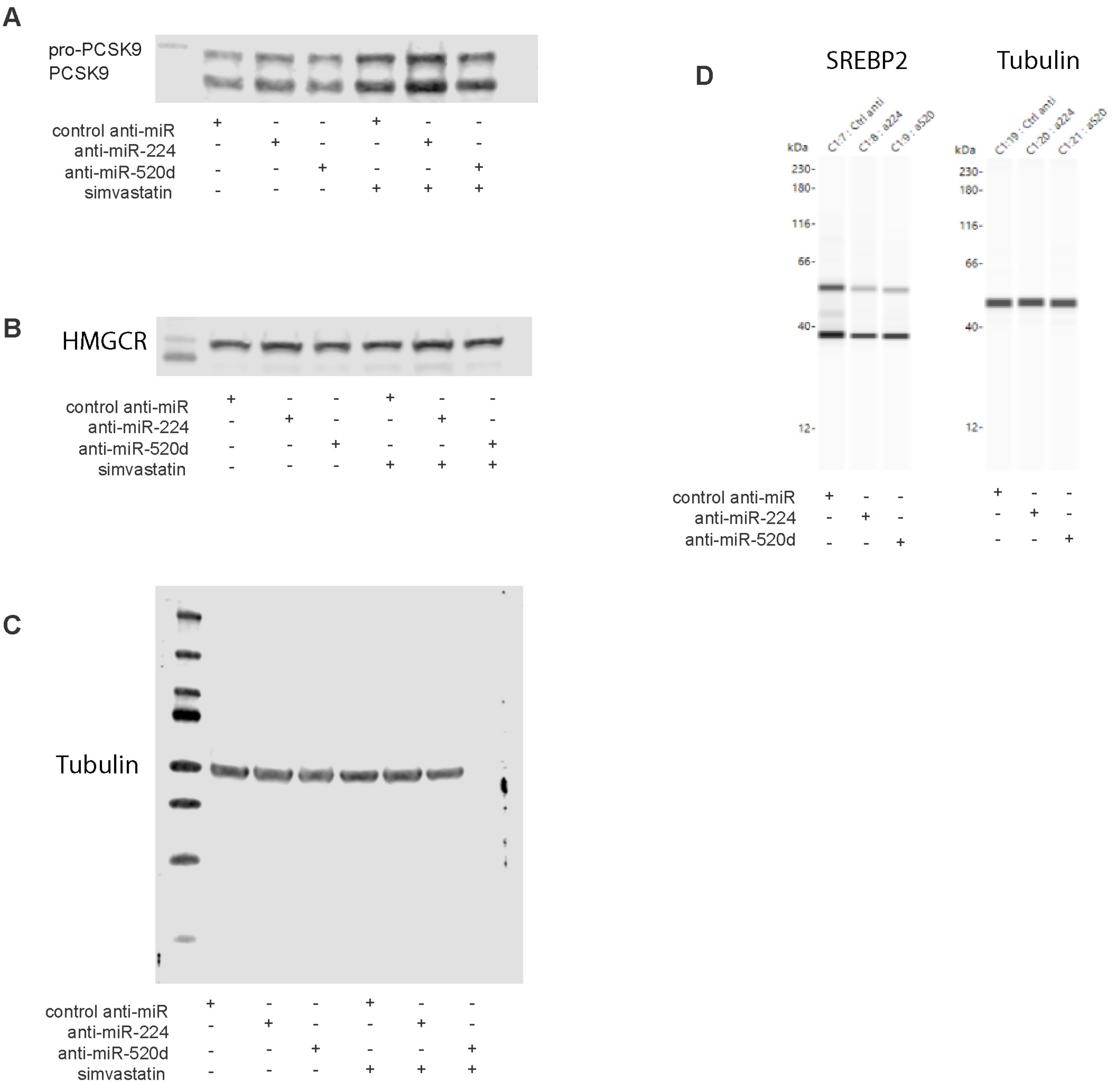

Supplemental Figure 8

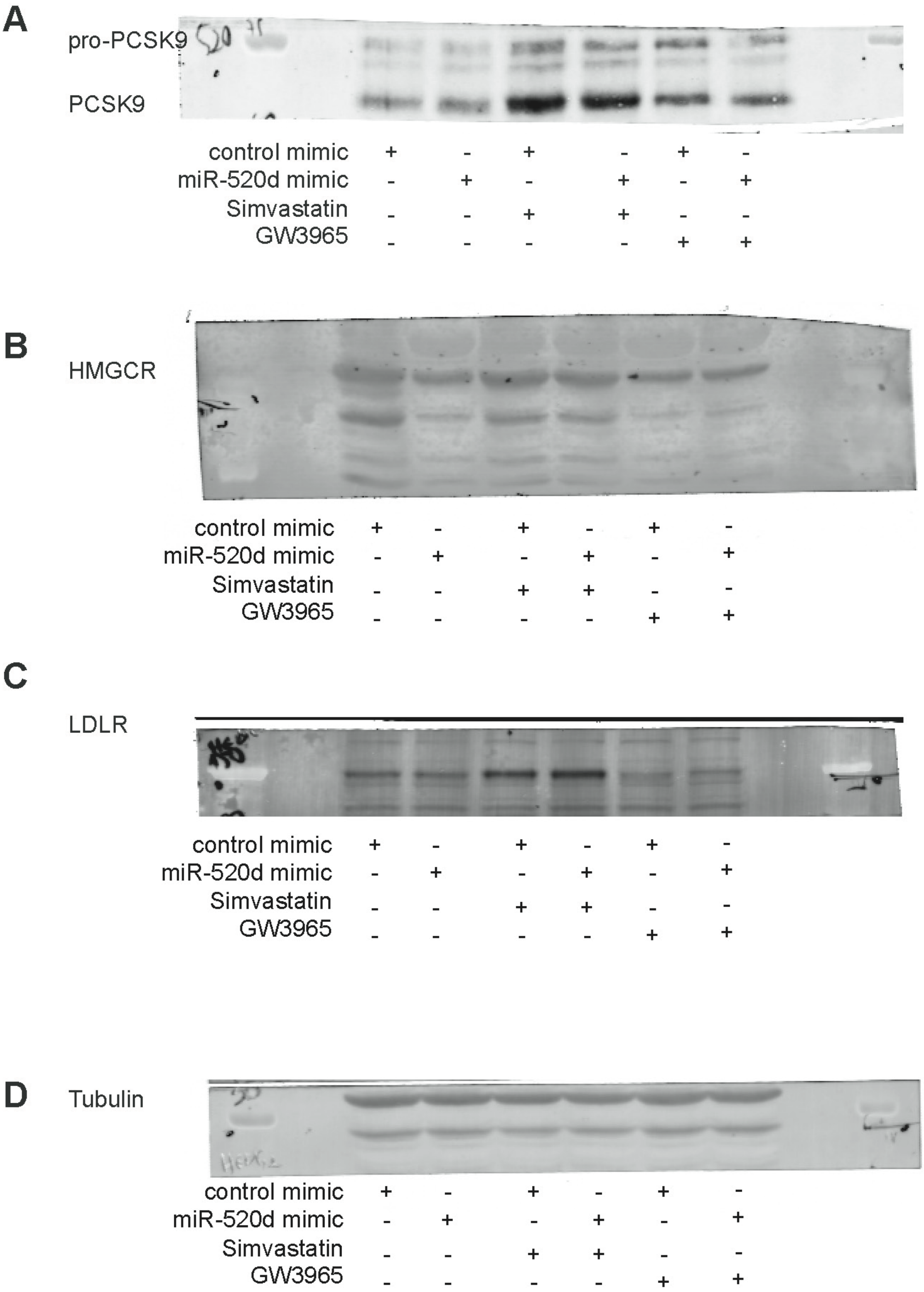

Supplemental Figure 9

A

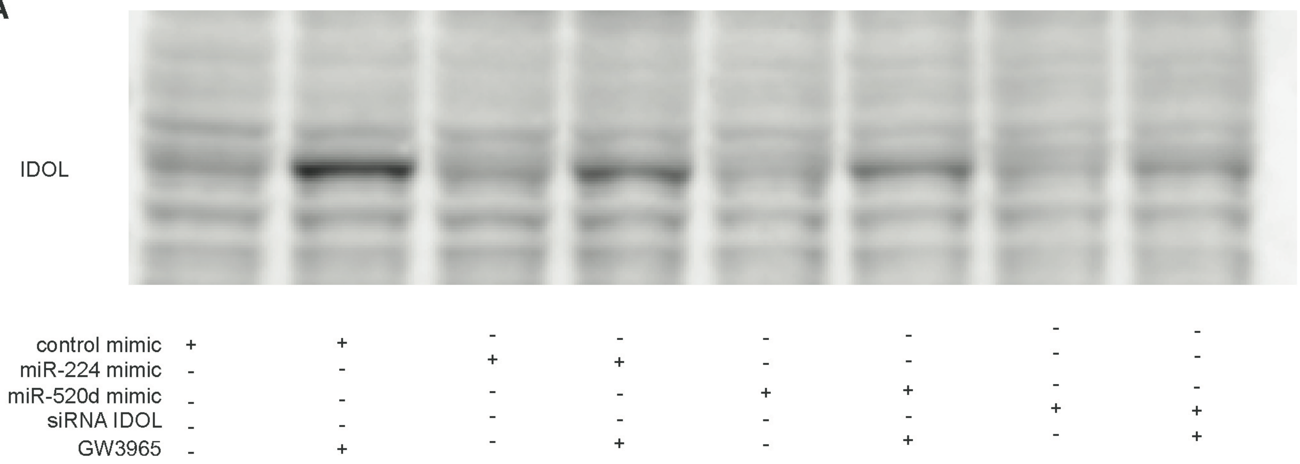

B

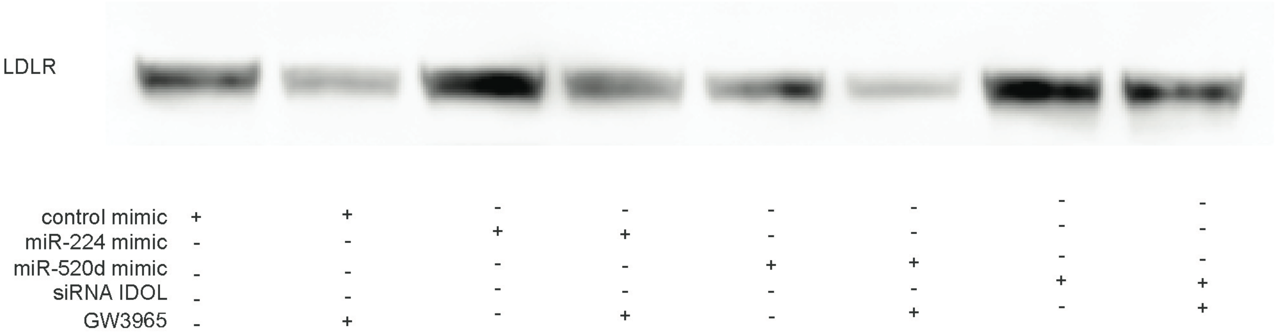

C

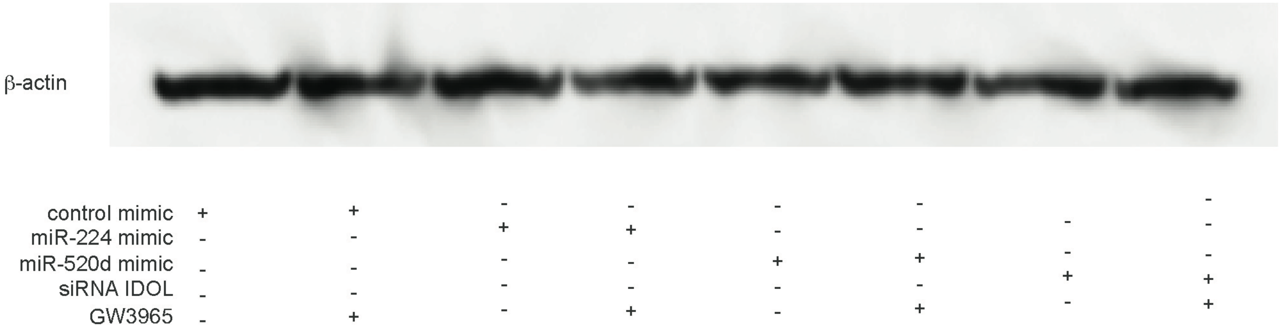

Supplemental Figure 10

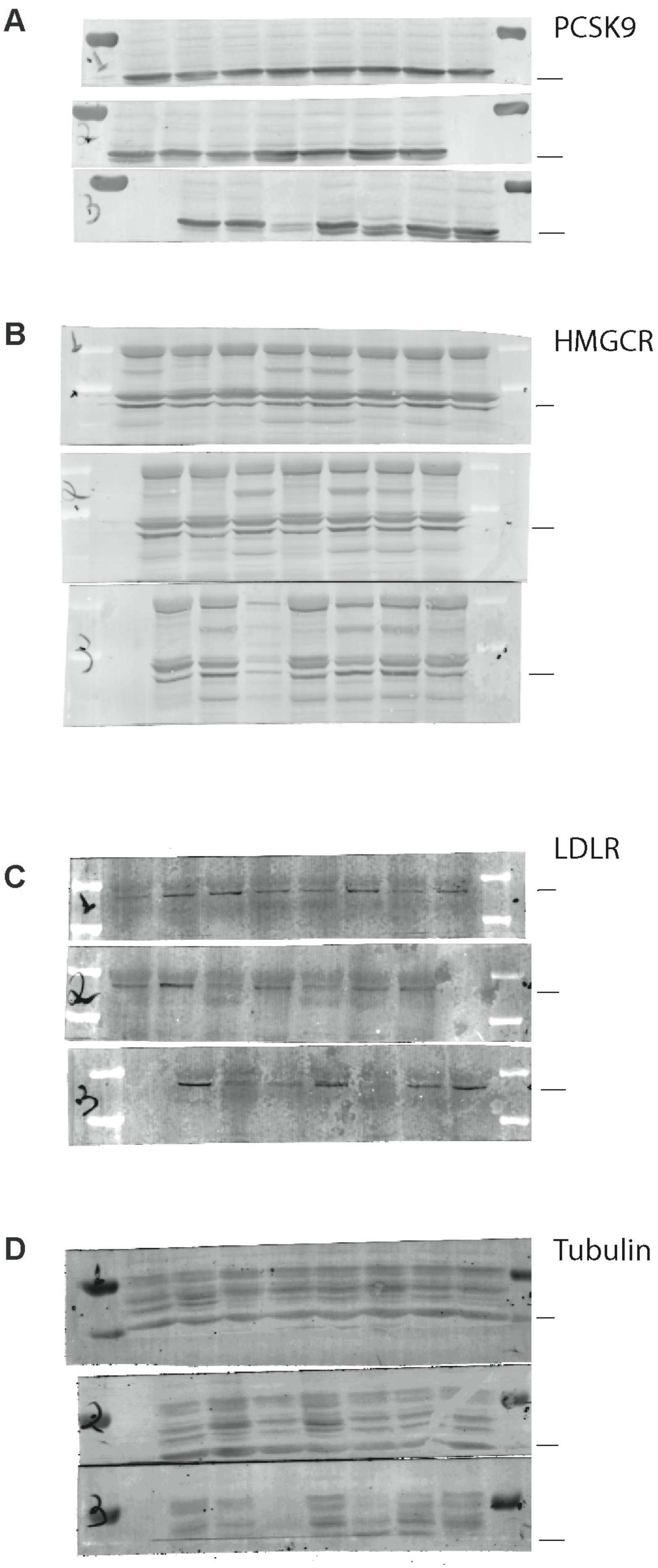

Supplement: Supplementary Figure 1 — Relative microRNA expression in cholesterol loaded vs. depleted macrophages. [file Data_Sheet_1.pdf]
